# Positive social modeling attenuates nocebo side effects

**DOI:** 10.1093/abm/kaaf048

**Published:** 2025-07-15

**Authors:** Cosette Saunders, Winston Tan, David Ng, Alexander Burchett, Nicolas McNair, Ben Colagiuri

**Affiliations:** School of Psychology, University of Sydney, Sydney, Australia; School of Psychology, University of Sydney, Sydney, Australia; School of Psychology, University of Sydney, Sydney, Australia; School of Psychology, University of Sydney, Sydney, Australia; School of Psychology, University of Sydney, Sydney, Australia; School of Psychology, University of Sydney, Sydney, Australia

**Keywords:** instruction, nocebo effect, placebo, social learning, social modeling, side effects

## Abstract

**Background:**

Receiving negative instructions and observing another’s adverse treatment-related experience can lead to worsened health outcomes via the nocebo effect. However, it is unknown whether the observation of a positive treatment-related experience can mitigate these effects.

**Purpose:**

To investigate whether a positive social modeling intervention can reduce nocebo side effects induced by instruction and social modeling.

**Methods:**

Participants (*N* = 160) were told the study assessed a new cognitive enhancer (actually a placebo). Participants received side effect warnings and viewed an informational video describing the medication. Placebo-treated groups were randomized to either watch an additional clip where a peer reported a positive experience with no side effects or not. These groups were further randomized to either encounter a live model exhibiting side effects or not. A Natural History group did not view any modeling nor receive the placebo. The primary outcome was the severity of side effects.

**Results:**

A significant nocebo effect was observed, with increased symptom severity in placebo-treated groups compared to the Natural History group. The positive social modeling intervention (i.e., viewing a peer experience no side effects) significantly reduced symptom severity. No significant difference in symptom severity was found between instruction alone and instruction with side effect modeling, nor was there an interaction between the induction method and the positive social modeling intervention.

**Conclusions:**

Positive social modeling reduces nocebo side effects induced by instruction alone and instruction with side effect modeling. Positive social modeling may be an effective method to mitigate the burden of nocebo side effects in clinical settings.

**Open Science Transparency Statements**

1. The study was pre-registered at AsPredicted.org: https://aspredicted.org/hk9p-h5tr.pdf
2. The analysis plan was registered prior to beginning data collection at AsPredicted.org: https://aspredicted.org/hk9p-h5tr.pdf
3. De-identified data from this study are available in a public archive: https://osf.io/zfas7/
4. Analytic code used to conduct the analyses presented in this study are available in a public archive: https://osf.io/zfas7/
5. Some of the materials used to conduct the study are presented in a public archive: https://osf.io/zfas7/

## Introduction

The nocebo effect, a pervasive psychobiological phenomenon in which individuals develop or experience an exacerbation of symptoms beyond what can be attributed to a treatment’s active elements, is increasingly recognized as a significant factor contributing to the experience of side effects. A large-scale review of pharmacological clinical trials involving over half a million participants found that nearly 3-quarters of placebo-treated individuals reported side effects and that these rates were often comparable with the drug arm.^1^ The time and cost associated with the management of side effects impose a substantial burden on our healthcare systems. In the US alone, managing adverse drug reactions is estimated to cost billions annually^2^ with side effects often leading to worse treatment adherence and even treatment cessation.^1^ Given the significant clinical and financial implications, identifying strategies to mitigate nocebo effects is critical.

In the absence of prior direct experience, nocebo effects can arise through instruction. Instruction refers to the information individuals receive about their treatment, such as when a health professional informs a patient that a medication may cause side effects (verbal) or reading the side effect information listed on medication packaging (written). While such side effect warnings are necessary for informed consent, a wealth of evidence indicates that they can produce negative expectancies that exacerbate side effects via the nocebo effect.^3–7^ For example, patients receiving analgesia found the injection more painful when told “You will feel a big bee sting; this is the worst part,” compared to more neutral wording.^8^

Research to date on strategies to minimize instructed nocebo effects is sparse and inconsistent. One proposed method is side effect framing, which involves presenting statistical information associated with side effects in a positive manner, e.g., “7 in 10 patients WILL NOT experience headaches compared to “3 in 10 patients WILL experience headaches.”^9^ Although Faasse et al.^10^ found that this approach significantly reduced the nocebo effect during a one-hour experimental session, no effect was present when assessed 24 hours later. Furthermore, other empirical studies fail to find any effect of side effect framing.^11,12^ Another suggested strategy is “nocebo education,” or informing patients about the nature and mechanisms of the nocebo effect.^13,14^ However, educational interventions can sometimes yield minimal benefits or even backfire.^15^ For example, one study aimed to improve perceptions of generic medicines via an educational video, but unexpectedly found reduced pain relief and increased symptoms when participants used the generic versus the branded version of the same medication.^15^ Although the intervention improved the perception of generic drugs, it may have unintentionally highlighted differences between generic and branded products or heightened participants’ attention to potential side effects—paradoxically worsening health outcomes, consistent with research concerning educational interventions with respect to other health outcomes.^16^ Given the substantial health and societal risks posed by the nocebo effect, it is crucial that novel and more effective strategies be investigated and implemented.

Importantly, in addition to instructions, we also acquire expectations via social learning. Social learning refers to what we learn by observing other’s experiences, such as observing a friend experience headaches after taking a medication, and subsequently expecting and experiencing increased headaches after our own encounter with the medication.^17^ Socially induced nocebo effects are robust and can influence a variety of symptom domains including pain, itch, nausea, and general side effects. Furthermore, recent evidence indicates that socially induced nocebo effects can be passed along social chains^18,19^ and can be triggered by exposure to social media.^20^ Concerningly, this research indicates that observing someone else experience a nocebo effect itself, can lead to the proliferation of nocebo effects. As such, social learning presents a potentially significant cumulative trigger for nocebo-induced side effects across society and health settings.

Yet, while socially induced nocebo effects are concerning, social learning may also provide an avenue to inhibit nocebo effects via positive social modeling. While placebo and nocebo effects do appear to have some important differences in their psychological and neurobiological mechanisms,^21^ the fact that positive social modeling induces placebo effects raises the possibility that positive social modeling could counteract the negative expectancies induced by nocebo instructions and social learning. Many nocebo social learning studies use a control group in which the model does not report any symptoms,^22^ which could be considered “positive” social modeling if the key outcome is the presence of side effects. Critically, however, in those studies participants are only ever exposed to either a negative or a positive social model and so they do not provide any evidence regarding whether positive social modeling can counteract nocebo effects.

To address this gap, the present study investigated whether a novel positive social information intervention could reduce nocebo side effects induced by instruction and social modeling. Participants were told they were part of a study investigating the efficacy of a new cognitive enhancement medication—which was actually a placebo. All participants received warnings about side effects and watched a short video concerning the supposed new cognitive enhancement medication. This medication was described to participants in the consent form and via the video as “associated with the experience of headaches and dizziness.” Participants in the Intervention condition viewed an additional clip where a social model (actually one of the researchers) reported a positive experience with the medication, finding it effective and communicating their lack of side effect experience. Participants in the No Intervention condition were not exposed to this positive social model. After the placebo was administered in the treatment groups, participants in the Instruction + Side effect Modeling condition then watched a live social model (a confederate participant) experience side effects from the medication. Participants in the Instruction Alone condition were not exposed to this negative social model. We hypothesized that there would be an overall nocebo effect, with increased symptom reporting in the placebo groups compared to the control group. Furthermore, we expected that live social modeling of side effects would exacerbate this nocebo effect compared to instruction alone. Most importantly, we hypothesized that positive social information would reduce nocebo side effects relative to conditions without such information. Finally, we predicted that the Positive Social Modeling Intervention would be more effective in participants exposed to negative social modeling than in those who received instruction alone, reflecting an interaction between side effect modeling and the positive social information intervention.

## Methods

The study design and analyses were pre-registered (aspredicted.org #163947). Ethics approval was granted by the University of Sydney (#2022/532).

**Figure 1.**
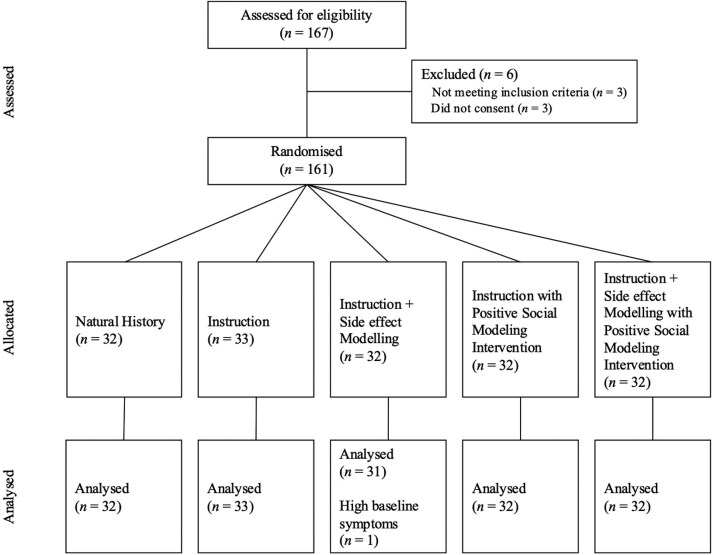
CONSORT flow diagram of participant selection process.

Participants. One hundred and sixty healthy volunteers (Female = 110, Male = 46, Other = 4, *M*_age_ = 20.16, *SD* = 3.97, Range 17 - 48) from the University of Sydney participated and received course credit as compensation over the period February–July 2024. Figure 1 presents a CONSORT Flow Diagram of participant selection. Information regarding sample race and socioeconomic status was not collected. Eligible participants were healthy adults fluent in English without any known allergy to medication or lactose. Due to the gelatin capsules, those with dietary restrictions were advised not to participate. To ensure participants were not experiencing significant symptoms at the time of testing, they were excluded from analysis if they exceeded pre-registered thresholds of physical symptoms pre-treatment (details below).

### Design

The study employed a single-blind between-subjects design with participants randomized using R 4.2.2^23^ as a random number generator to 1 of 5 conditions based on a 2 (Nocebo Induction Method: Instruction Alone vs. Instruction + Side effect Modeling) × 2 (Positive Social Modeling Intervention: Positive Model vs. No Positive Model) factorial design with an additional Natural History control group. Participants were told the study was investigating the efficacy of a new cognitive enhancer (actually a placebo). All participants then viewed one of 2 versions of a 5-minute informational video. In the No Intervention condition, the video featured a researcher describing the supposed cognitive enhancer’s benefits and potential side effects (i.e., headaches & dizziness), along with a demonstration of the general procedure using footage of a supposed previous participant (actually a confederate). In this version of the video the confederate was only seen up to the point of taking the treatment, so they did not provide any information about the treatment experience or side effects. In the Intervention condition, the same video was presented but it also included an additional 30-second segment at the end, where the supposed previous participant was asked about the cognitive enhancer’s efficacy and side effects and explicitly reported the absence of any side effects. The videos are publicly available on OSF https://osf.io/zfas7/. Participants were then further randomized to either encounter a live social model who reported side effects (“Instruction + Side effect Modeling”) or not (“Instruction Alone”). This model was a different confederate presented to participants as another participant of the study who was concluding their participant time such that the model was asked about side effects and verbally reported experiencing headaches and dizziness. This led to 4 groups: Instruction, Instruction with Positive Social Modeling Intervention, Instruction + Side effect Modeling, and Instruction + Side effect Modeling with Positive Social Modeling Intervention. A fifth group was included to create a natural history/control condition to enable an overall assessment of the nocebo effect. This group viewed the “No Intervention” version of the informational video but did not receive the placebo treatment. The primary dependent variable was the severity of symptoms reported by participants.

### Materials and measures

#### Physical symptoms.

Physical symptoms were assessed using a 10-item modified version of the General Assessment of Side Effects, GASE.^24^ Each of the 10 symptoms assessed was rated using a 7-point scale 0 (not present) to 7 (severe). The primary outcome of interest was sum-score of the headaches and dizziness items from the GASE corresponding to the side effect warnings and symptoms modeled by the live model. In accordance with pre-registration, those with extreme baseline side effects (6 or more on any single item, or a mean greater than 4) were excluded from analyses.

#### Symptom expectancy.

Participants were asked to rate their expectancy of the experience of side effects on the single item measure: “How much do you expect to experience adverse events (e.g., side effects) as a result of participating in this study?” on a Visual Analogue Scale (VAS) ranging from 1 (not at all) to 100 (very much so).

#### Expectancy for cognitive enhancement.

Participants were asked to rate their expectancy of the efficacy of the cognitive enhancement medication on the single item measure: “To what degree do you expect the cognitive enhancement medication to enhance your performance?” on a VAS ranging from 1(No enhancement) to 100(Significant enhancement).

#### Generalized state anxiety.

General state anxiety was measured via the Spielberger State-Trait Anxiety Inventory-6 (Cronbach’s alpha = .75).^25^ Participants rated 6 items like “I am relaxed” on a 4-point scale ranging from 1 (not at all) to 4 (very much) based on how they felt at the present.

#### Side effect specific anxiety.

Participants rated their anxiety concerning experiencing side effects on the single item measure: “How anxious are you about experiencing adverse events (e.g., side effects) as a result of participating in this study?” on a VAS ranging from 1(Not Anxious) to 100(Very Anxious).

#### Objective cognitive performance.

Sustained attention was measured using the Rapid Visual Information Processing (RVIP) task.^26^ For 5 minutes, numbers from 1 to 9 were displayed at a speed of 100/min on a computer screen. Participants were instructed to press the space bar when 3 consecutive even or 3 consecutive odd numbers appeared in the sequence. They had 1.5 seconds to respond appropriately, or the response was regarded as a false alarm. The target sequences were spaced by a minimum of 5 and a maximum of 33 digits due to the semi-random nature of the number series. A performance evaluation was conducted using the percentage (%) of correct answers.

#### Self-reported cognitive performance.

Participants were asked to rate their perceived performance on the RVIP: “How would you rate your performance on the cognitive task?” on a VAS ranging from 1(Very poor) to 100(Very good).

#### Self-reported impact of treatment on cognitive performance.

Participants assigned to take the placebo were asked “How effective was the medication at enhancing your cognitive performance?” on a VAS ranging from 1(Not effective at all) to 100(Very effective).

#### Manipulation check.

Participants were asked “Briefly describe (in 2-3 sentences) what you thought the purpose of the experiment was”. The probe was intentionally vague to prevent prematurely raising suspicion regarding the study within the student cohort.

#### Placebo capsules.

All participants assigned to take the “cognitive enhancer” received white and blue gelatin capsules filled with lactose.

#### Heart rate and electrodermal activity.

Participant heart rate and electrodermal activity were measured. Details can be found in Supplementary Materials 6.

#### Laboratory setting.

The lab was set up like a clinic room to reinforce the cover story. The room contained a medical examination bed and potted plants. The walls were decorated with posters about the anatomy of the brain, memory processes, and attention. The researcher wore a lab coat, and a heart rate monitor was visibly used, all reinforcing the realism of an experimental medication study.

### Procedure

**Figure 2.**
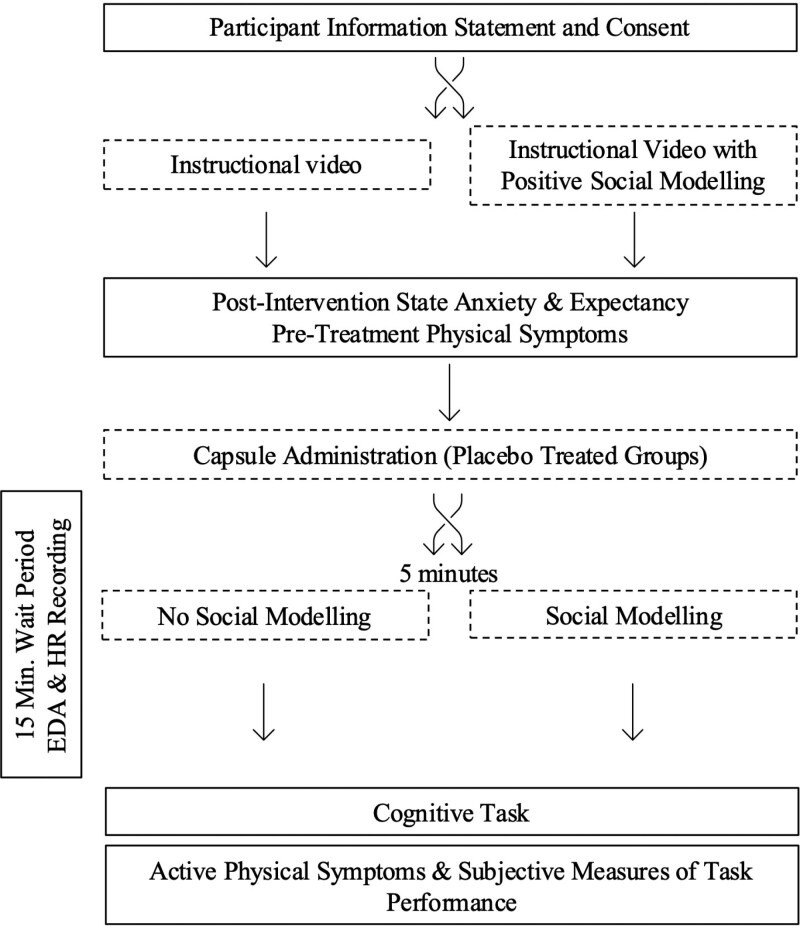
Flowchart of study procedure. All participants underwent steps outlined by solid boxes. Boxes with dashed lines indicate a step participants were randomized to undertake. Participants in the Natural History group watched the instructional video with no positive social modeling, did not receive the placebo treatment and did not view the live side effect modeling.

Refer to Figure 2 for a flow chart of the study procedure. Participants each attended a 1-hour experimental session. Participants first received an information statement and consent form that outlined the cover story and contained the side effect warning concerning the supposed medication: “*Vitatril* has been associated with the experience of mild headaches and dizziness.” Once consent was obtained, participants were asked to watch a video described as a way to inform them of the study aim and procedures. The positive social modeling was implemented via this video. In the No Intervention and Natural History conditions the video detailed: the supposed medication, its mechanism of action, its side effects, and a demonstration of the study procedure with another participant (actually another researcher). Those assigned to the Intervention conditions, watched the same video as those in the No Intervention and Natural History conditions, with an additional scene at the end where the supposed participant was asked how they were feeling. The participants indicated that they felt the positive effects of the medication (i.e., “more focused”) and emphasized they did not feel any side effects. The videos are publicly available at https://osf.io/zfas7/. Participants were then fitted with the Equivital Harness and were asked to complete the demographic questions and post-intervention state anxiety and expectancy measures. In addition, a pre-treatment measure of physical symptoms was recorded to identify those who met the exclusion criteria and to control for symptoms being experienced at baseline. Next, participants were seated on a physical exam bed and set up with the electrodes to measure their EDA. The placebo capsules were then administered to treatment groups. All participants were seated and waited for 15 minutes, ostensibly providing time for the medication to take effect in the treatment groups. Exactly 5 minutes after the physiological recordings commenced, participants in the side effect modeling conditions saw a confederate enter the room. In view of the participant, the researcher asked the confederate how they were feeling and the confederate responded with “Not great, definitely feeling headachy… and a bit dizzy.” To account for the potential confound of having 2 different individuals as the positive social model and the side effect model respectively (as necessitated by study design), we counterbalanced which confederate served as the live and video models. Further, to control for gender, age and race, both confederates were Asian Australian men in their 20s. At the timepoint of side effect modeling in the Instruction + Side effect Modeling groups, participants in the other groups overhead the researcher have an irrelevant phone call to control for any generic effects of hearing a conversation. After the 15-minute wait period, all participants completed the cognitive task to uphold the cover story. Next, participants completed the active physical symptoms questionnaire, reported their perceived efficacy of the medication, and completed the manipulation check. At the conclusion of data collection, all participants received a written debrief informing them about the true aims of the study.

### Power and data analysis

All data analysis was conducted in R 4.2.2^23^ using a threshold of α < .05 to determine significance. The primary data analysis consisted of a 2 (Nocebo Induction Method: Instruction Alone, Instruction + Side effect Modeling) × 2 (Intervention: No Intervention, Intervention) + 1 (Natural History) ANOVA, using the symptom difference score (active - baseline/pre-treatment) as the dependent variable. Orthogonal contrasts were used to determine the presence of a nocebo effect (Placebo Treatment vs Natural History), the main effect of Nocebo Induction method (Instruction Alone vs Instruction + Side effect Modeling), the main effect of the Positive Social Modeling Intervention (Positive Social Modeling vs No Positive Social Modeling), and the interaction between the Positive Social Modeling Intervention and the Nocebo Induction Method. Secondary analysis explored side effect generalization with the remaining 8 GASE items sum-scored as the outcome variable, in analyses mirroring those detailed above. Moderation analyses were then conducted to explore group differences revealed in the primary analyses using gender, state anxiety, side effect anxiety, side effect expectancy as moderators. Self-reported cognitive performance and actual cognitive performance were compared between groups in a similar way to the primary analyses to assess the presence of a placebo effect. The effect of the Positive Social Modeling Intervention on expectancy and anxiety was assessed using t-tests comparing the groups that received the intervention to those that did not, collapsed across side effect modeling conditions as expectations and anxiety were measured prior to the side effect modeling manipulation. Expectations of side effects that might occur from the supposed treatment were assessed in all participants after the information about the study was delivered, which included the warning about side effects and Positive Social Modeling Intervention in the relevant groups, but before participants knew whether or not they would receive the supposed treatment (i.e., placebo capsules). Mediation analyses were therefore conducted to assess if the effect of the Positive Social Modeling Intervention on side effects was mediated via expectancies. Note, however, that since capsule administration in placebo treatment groups occurred after expectations were measured, we were unable to conduct a mediation to assess if the overall nocebo effect (Placebo Treatment vs Natural History) was mediated via expectancies.

As per pre-registration, a minimum of 32 participants per-group were recruited. This was based on a power analysis assuming a medium effect size for the Positive Social Modeling Intervention (f = .25, in the absence of prior research to inform the effect size) with an alpha of .05 with the power to detect an effect set at 80%.

## Results

### Demographic data

There were no significant differences between groups in age and gender (all *p≥*.291) indicating randomization was successful. See Electronic Supplementary Material 1.

### Main analysis (pre-registered)

#### Side effects.

**Figure 3.**
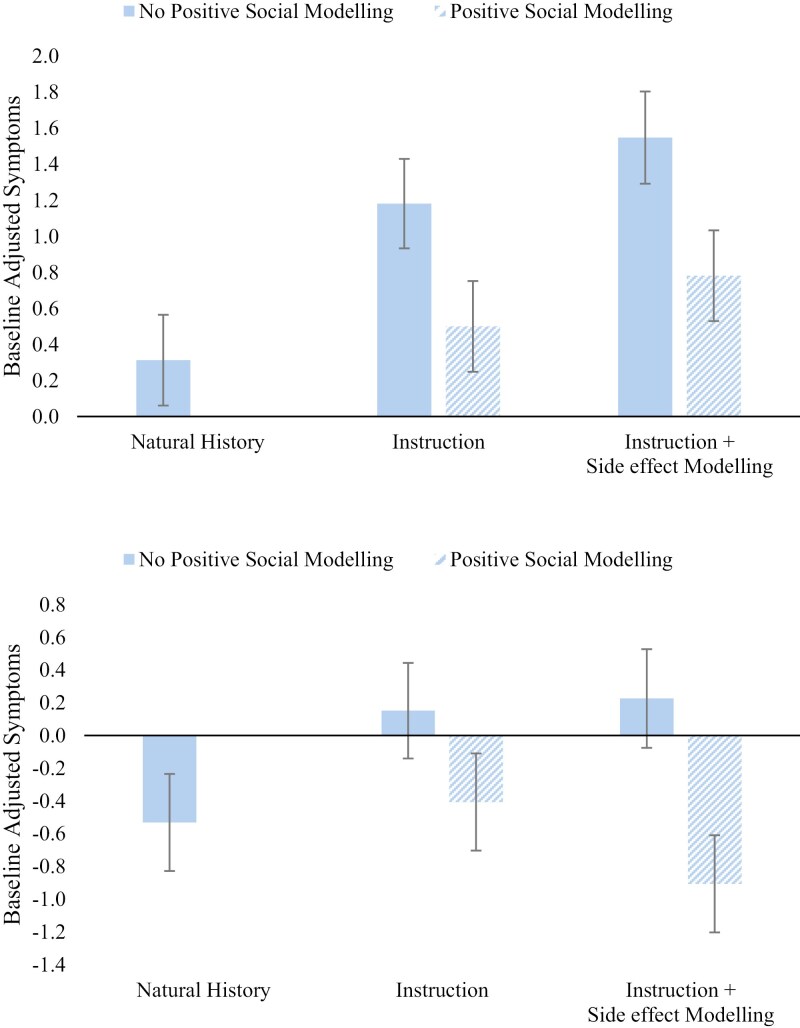
Baseline adjusted symptoms for each experimental condition. Group means for warned symptoms (top) and non-warned symptoms (bottom). All error bars are ± 1 Standard Error of the Mean (SEM).

Figure 3 depicts the group means for the ANOVA assessing group differences in reported side effect severity. Full statistics are available in the Electronic Supplementary Material 2. Orthogonal contrasts revealed that there was a significant overall nocebo effect, where groups that received the placebo reported increased severity of symptoms relative to the Natural History group, *F*(1,155) = 6.00, *p* = .015, *η*_*p*_^2^ = .038. There was no significant difference in symptom severity by induction method, *F*(1,155) = 1.65, *p* = .201, *η*_*p*_^2^ = .011. The positive modeling significantly decreased severity of symptoms reported, *F*(1,155) = 8.26, *p* = .005, *η*_*p*_^2^ = .051. There was no significant interaction between induction method and the effect of the positive social modeling *F*(1,155) = 0.03, *p* = .866, *η*_*p*_^2^ < .001. The lack of main effect of induction method may have been due to the efficacy of the intervention. To investigate this possibility, a simple effect examined whether induction method had significant effects for those that did not receive the positive modeling intervention, and this revealed no significant effect of induction method *F*(1,62) = 0.82, *p* = .369, *η*_*p*_^2^ = .013.

#### Generalization of side effects.

Figure 3 also depicts the group means for the ANOVA assessing group differences in reported side effect severity of all other symptoms assessed by the GASE. Full statistics are available in the Electronic Supplementary Material 3. The analysis revealed that there was no significant overall effect of the nocebo treatment on other symptoms, *F*(1,155) = 0.81, *p* = .371, *η*_*p*_^2^ = .005, and no significant main effect of social induction method, *F*(1,155) = 0.52, *p* = .474, *η*_*p*_^2^ = .003. There was a significant effect of the intervention on reducing other symptom’s severity, *F*(1,155) = 8.12, *p* = .005, *η*_*p*_^2^ = .050. Finally, the interaction between social induction method and the positive intervention was not significant, *F*(1,155) = 0.937, *p* = .334, *η*_*p*_^2^ = .006.

#### Moderation analysis for side effects.

Moderation analyses exploring the significant nocebo effect and significant effect of the Positive Social Modeling Intervention are presented in Table 1.

**Table 1.** Moderation analyses of the nocebo effect and the effect of the positive social modeling intervention.

	Nocebo			Intervention		
	*F*(df1,df2)	*P*	*η*_p_^2^	*F*(df1,df2)	*P*	*η*_p_^2^
Gender	0.06 (1,152)	.814	<.001	0.05 (1,120)	.831	<.001
STAI-6	0.21 (1,156)	.649	.001	2.02 (1,124)	.157	.016
Expectancy	0.32 (1,156)	.572	.003	4.50 (1,124)	.036	.035
Anxiety	0.07 (1,156)	.793	<.001	1.05 (1,124)	.307	.007

STAI-6 refers to the State-Trait Anxiety Inventory-6.^25^ Analyses involving gender have a sample size *N* = 156, excluding those who did not identify with either male or female due to insufficient sample size.

#### Cognitive performance.

**Figure 4.**
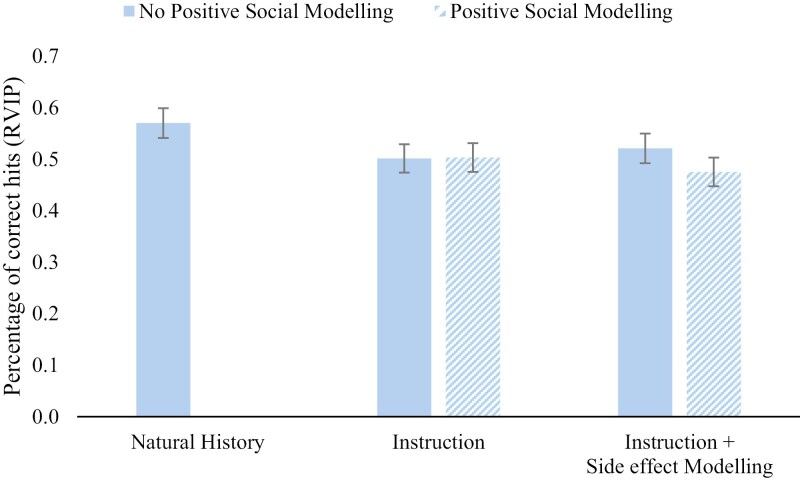
Group means of objective performance on the RVIP task.

Refer to Figure 4 for group means of objective cognitive performance. A significant reduction in objective cognitive performance was found in the treatment groups compared to the natural history group, *F*(1,152) = 4.75, *p* = .030, *η*_*p*_^2^ = .030. However, there was no significant effect of side effect modeling, *F*(1, 152) = 0.02, *p* = .880, *η*_*p*_^2^ < .001, the Positive Social Modeling Intervention, *F*(1, 152) = 0.62, *p* = .434, *η*_*p*_^2^ = .004, nor an interaction, *F*(1, 152) = 0.72, *p* = .400, *η*²=.005 on objective cognitive performance. Orthogonal contrasts revealed no significant differences between groups in self-reported cognitive performance all *p*>=.316. Within the 3 groups that received the placebo treatment, there was no significant difference in self-reported influence of treatment on cognitive performance, *F*(3, 124) = 0.51, *p* = .677, *η*²=.012. See Electronic Supplementary Materials 5 for full comparisons and group means.

### Exploratory analysis

#### Effects of the intervention on expectancy and anxiety.

**Figure 5.**
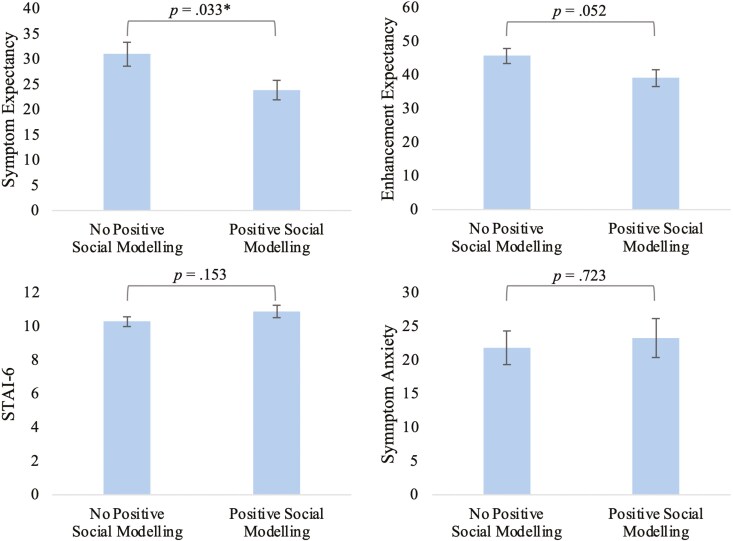
Comparison of anxiety and expectations between groups that received the Positive Social Modeling intervention and the groups that did not. * indicates *p* < .05, ** indicates *p* < .01, *** indicates *p* < .001.

T-tests were used to compare expectations for side effects, expectations for cognitive enhancement, side effect anxiety and state anxiety between groups that received the Positive Social Modeling Intervention (i.e., Instruction with Intervention and Instruction + Side effect modeling with Intervention) versus that those who did not (i.e., Natural History, Instruction Alone and Instruction + Side effect modeling). As shown in Figure 5, the intervention significantly decreased expectancies for symptoms, *t*(158) = -2.15, *p* = .033, *d* = -0.35, but not expected cognitive enhancement, *t*(158) = -1.95, *p* = .052, *d* = -0.32. The Intervention did not significantly affect state anxiety, *t*(158) = 1.44, *p* = .153, *d* = 0.23, nor symptom-specific anxiety, *t*(158) = 0.35, *p* = .723, *d* = 0.06.

#### Mediation of the intervention effect on side effects by expectations.

 As there were effects of the intervention on both symptom expectancy and symptom severity, a mediation analysis was conducted to investigate if the effect of the intervention on the nocebo effect was mediated by expectations. The independent variable was Intervention: Intervention vs No Intervention, collapsed across induction method, excluding the Natural History group. The dependant variable was symptom severity and expectancy was the mediator. Bootstrapping with 10,000 samples was conducted to determine 95% confidence intervals (CIs) which were used to determine significance. Expectancy did not significantly mediate the effect of the intervention on symptom severity, direct effect = 0.70, 95% CI [0.20,1.22], *p* = .008 and indirect effect = 0.02, 95% CI [-0.05,0.16], *p* = .671.

## Manipulation check

Both social modeling manipulations were successful, no participants reported suspicion regarding the positive social modeling via the instructional video and only 2 participants in the sample reported suspicion concerning the live social model. A small proportion of participants expressed suspicion that the treatment was a placebo (*N* = 21, 13%). However, raising suspicion concerning the placebo treatment did not differ statistically between groups, χ²(4, *N* = 160) = 3.96, *p* = .412, Cramer’s V = .16, nor was it associated with a change in target symptom reporting, controlling for group, *F*(1, 150) = 1.76, *p* = .187, *η*_*p*_^2^ = .012.

## Discussion

This was the first study to investigate whether positive social modeling, i.e., modeling a lack of side effects, could reduce nocebo side effects. As anticipated, we found clear evidence of a nocebo effect, with participants in the (placebo) treatment groups reporting significantly greater side effect severity compared to the Natural History group. Contrary to our hypotheses, the combination of side effect modeling with a side effect warning did not increase nocebo side effects relative to the warning without side effect modeling. Importantly, the novel positive social modeling intervention effectively reduced nocebo symptom severity across treatment groups, demonstrating its utility in mitigating nocebo effects. There was no significant interaction between the nocebo induction method and the positive social modeling intervention, indicating that the intervention’s effectiveness was consistent regardless of the method of nocebo induction. These findings have several important theoretical and clinical implications.

Most importantly, the present study found that providing participants with positive social modeling inhibited nocebo side effects irrespective of whether the nocebo effect was induced by instruction alone or instruction and side effect modeling, and this positive effect extended to both other (non-warned) side effects. Positive social modeling has previously been found to lead to placebo effects,^27,28^ but to the best of our knowledge has never previously been examined as a potential preventive strategy to combat nocebo effects. Two aspects of this finding are particularly interesting. The first is that the positive modeling was video-based whereas the side effect modeling was in person. A recent meta-analysis revealed that, typically, in person modeling produces stronger effects than video-based modeling.^22^ As such, it is particularly noteworthy that the video-based positive modeling effectively inhibited the nocebo effect even when it involved verbal instruction accompanied by in person side effect modeling. This suggests that video-based positive modeling could be an effective and scalable technique for combatting nocebo effects even when they involve in person observation of another person experiencing adverse symptoms from a treatment. Second, the fact that positive social modeling inhibits nocebo effects means that commonly used “neutral” control groups in social modeling nocebo research^22^ may actually be inhibitory if they involve communication indicating that no adverse symptoms occurred following the treatment, whether direct (as in the current study) or indirect (e.g., absence of a report of side effect). As such, estimating the magnitude of any socially induced nocebo effect might be more accurate when a true natural history group is used that is not exposed to any social modeling.

Related to this, it was interesting to observe that contrary to predictions, the addition of side effect modeling did not significantly exacerbate the nocebo effect induced via instruction alone. This finding appears inconsistent with a recent meta-analysis demonstrating that instruction with social modeling typically produced larger nocebo effects than verbal instruction alone does.^22^ However, it is important to note that the current study employed a more formal verbal instruction than is typically used in other studies in that, in addition to being written in the consent form, the side effect warning was presented as part of an explanatory video provided to the participants about the supposed medication and trial. In comparison, in many warning-induced nocebo studies side effect information may only be presented in written materials like the consent form or through brief verbal discussion ^[e.g.,^.^4,29–31]^ The use of an explanatory video in the current study may have increased the instructed nocebo effect and created a ceiling effect that reduced the impact of the addition of the side effect social modeling. Supporting this, the effect size of the instructed nocebo effect in the current study was approximately 50% larger than the typical instructed nocebo effect observed in nocebo research.^32^ It would therefore be interesting for future research to compare the effect of the addition of social modeling to side effect warnings with varying strengths of instructions.

Expectancy and anxiety are key factors hypothesized to facilitate nocebo effects.^33^ The positive social modeling video intervention successfully reduced expectations for symptoms, however, did not significantly affect participant anxiety. Although symptom expectancy was reduced because of the intervention, side effect expectations did not mediate the reduction in the nocebo effect caused by the intervention. This may suggest that other unmeasured factors are responsible for the intervention’s effectiveness. Alternatively, it could be that the timing of the expectancy measurement resulted in a less sensitive measure and that this undermined evidence of mediation. Symptom expectancy was assessed immediately after the intervention, but prior to placebo administration, potentially making it an outdated measure if there are expectancies that arise once the participant is informed they are assigned to take or not take the supposed “cognitive enhancer,” and from the treatment administration process itself. Additionally, the 15-minute wait period allows ample time to reflect upon and contemplate the information concerning the “treatment” that the participant received. While we believe it was not feasible in this study to measure side effect expectancy repeatedly without revealing the study’s true nature, future research should incorporate expectancy measurements after group allocation and following social modeling manipulations to better understand the role of expectancy in nocebo effects.

Interestingly, we observed no placebo-related improvement on either subjective or objective measures of cognitive performance and in fact, those who received the placebo demonstrated worsened objective cognitive performance. Where previous research has reported placebo cognitive enhancement^34–36^ the broader study contexts have focused on investigating the primary effect of the placebo (i.e., the cognitive enhancement) with little to no emphasis on side effects. The focus of the current study was on side effects so it may be the case that the instructions delivered concerning cognitive enhancement were less strong than in other studies for which it’s a primary outcome. It is also possible that the experience of side effects due to the nocebo effect in the treatment groups may have engendered discomfort or distraction sufficient to impair cognitive functioning—a hypothesis that warrants investigation in future studies.

Gender was not found to moderate the strength of the nocebo effect, nor influence the efficacy of the intervention. Previous research has found that female observers can be more susceptible to nocebo effects under certain conditions,^37,38^ however others suggest it maybe the model’s gender—or a match between model and observer—that primarily drives observed differences.^22,39^ In the present study, both the side effect social model and positive social model were male, leaving questions about the precise role of model and observer gender an avenue for future investigation.

Some limitations to the study are worth discussing. First, the study was conducted with healthy psychology undergraduates and therefore would benefit from replication in clinical settings to investigate if the intervention’s benefits translate in clinical practice. Interestingly, the nocebo effect tends to be *larger* in clinical populations,^32,40^ and factors specific to the clinical environment like presence of other patients and disease comorbidities may influence the nocebo effect and its mitigation in ways not captured here. Additionally, the study did not collect data on participant ethnicity or socioeconomic status, limiting our ability to comment on the generalisability of our findings to the broader population. Second, the study was conducted over the span of one hour, meaning it is unknown how long the positive effects of the intervention can be sustained. Third, several of the measures (e.g., side effect anxiety and expectancy) were single-item scales that have not been extensively validated in previous research; although they were brief and aligned well with our specific focus, it would be beneficial to replicate our findings with validated instruments. Finally, To translate this intervention into a feasible and useful clinical tool it is crucial to investigate patient acceptability of this intervention. This would indicate whether the positive social modeling is perceived by patients as acceptable and ethical.

In conclusion, this study highlights the role that nocebo effects can play in generating side effects and importantly provides novel evidence that positive social modeling may be a way of combatting these effects. The efficacy of positive social modeling here is particularly noteworthy given that it was video-based and was effective even for a combination of nocebo instructions and in person side effect modeling. This suggests that positive social modeling may be an effective and scalable method to reduce the significant burden nocebo effects cause. Future research is needed to translate this finding to clinical settings as well as to examine patient perspectives on its acceptability.

## Supplementary Material

kaaf048_suppl_Supplementary_Materials_1-6
