# Supplementary material for: Positive social modeling attenuates nocebo side effects
Source: Ann Behav Med. 2025 Jul 15;59(1):kaaf048. doi: 10.1093/abm/kaaf048 (PMC12260372; doi:10.1093/abm/kaaf048)
Supplement: kaaf048_suppl_Supplementary_Materials_1-6 [file kaaf048_suppl_supplementary_materials_1-6.docx]

**Supplementary Materials**

All analytic code and further results can be found at OSF <https://osf.io/zfas7/>

**Supplementary Material 1. Demographic Data**

## *ANOVA (Age ~ Group)*

|  | **Sum Sq** | **Df** | **F value** | **Pr(>F)** |
| --- | --- | --- | --- | --- |
| (Intercept) | 65026.56482 | 1 | 4152.18860 | 0.00000 |
| group | 78.35213 | 4 | 1.25077 | 0.29188 |
| Residuals | 2427.42287 | 155 | NA | NA |

| **group** | **emmean** | **SE** | **df** | **lower.CL** | **upper.CL** |
| --- | --- | --- | --- | --- | --- |
| NH | 20.21875 | 0.69957 | 155 | 18.83683 | 21.60067 |
| NINSM | 19.48485 | 0.68889 | 155 | 18.12402 | 20.84567 |
| INSM | 21.46875 | 0.69957 | 155 | 20.08683 | 22.85067 |
| NISM | 19.67742 | 0.71076 | 155 | 18.27338 | 21.08146 |
| ISM | 19.96875 | 0.69957 | 155 | 18.58683 | 21.35067 |

## *Gender (N)*

|  | **NH** | **NINSM** | **INSM** | **NISM** | **ISM** |
| --- | --- | --- | --- | --- | --- |
| Female | 23 | 24 | 23 | 18 | 22 |
| Male | 9 | 8 | 8 | 12 | 9 |
| other | 0 | 1 | 1 | 1 | 1 |

**Supplementary Material 2. Pre-registered Analysis: Side effects**

*ANOVA (Primary side effects ~ Group)*

|  | **Sum Sq** | **Df** | **F value** | **Pr(>F)** |
| --- | --- | --- | --- | --- |
| (Intercept) | 119.61140 | 1 | 58.86944 | 0.00000 |
| group | 32.04474 | 4 | 3.94288 | 0.00447 |
| Residuals | 314.93026 | 155 | NA | NA |

|  | **eta.sq** | **eta.sq.part** |
| --- | --- | --- |
| group | 0.09235 | 0.09235 |

**Supplementary Material 3. Generalisation of side effects**

*ANOVA (Other side effects ~ Group)*

|  | **Sum Sq** | **Df** | **F value** | **Pr(>F)** |
| --- | --- | --- | --- | --- |
| (Intercept) | 13.75726 | 1 | 4.89001 | 0.02848 |
| group | 29.12572 | 4 | 2.58818 | 0.03901 |
| Residuals | 436.06803 | 155 | NA | NA |

|  | **eta.sq** | **eta.sq.part** |
| --- | --- | --- |
| group | 0.06261 | 0.06261 |

| **group** | **emmean** | **SE** | **df** | **lower.CL** | **upper.CL** |
| --- | --- | --- | --- | --- | --- |
| NH | -0.53125 | 0.29651 | 155 | -1.11697 | 0.05447 |
| NINSM | 0.15152 | 0.29198 | 155 | -0.42526 | 0.72829 |
| INSM | -0.40625 | 0.29651 | 155 | -0.99197 | 0.17947 |
| NISM | 0.22581 | 0.30125 | 155 | -0.36928 | 0.82090 |
| ISM | -0.90625 | 0.29651 | 155 | -1.49197 | -0.32053 |

## **Supplementary Material 4. Moderation analyses**

*Moderation analyses: Gender * Nocebo effect*

|  | **Sum Sq** | **Df** | **F value** | **Pr(>F)** |
| --- | --- | --- | --- | --- |
| (Intercept) | 90.69960 | 1 | 41.65812 | 0.00000 |
| treat | 11.07577 | 1 | 5.08708 | 0.02553 |
| gender | 0.80632 | 1 | 0.37034 | 0.54373 |
| treat:gender | 0.12143 | 1 | 0.05577 | 0.81362 |
| Residuals | 330.94000 | 152 | NA | NA |

|  | **eta.sq** | **eta.sq.part** |
| --- | --- | --- |
| treat | 0.03218 | 0.03238 |
| gender | 0.00234 | 0.00243 |
| treat:gender | 0.00035 | 0.00037 |

*Moderation analyses: Gender * Intervention effect*

|  | **Sum Sq** | **Df** | **F value** | **Pr(>F)** |
| --- | --- | --- | --- | --- |
| (Intercept) | 98.50784 | 1 | 42.63673 | 0.00000 |
| intervention | 11.79430 | 1 | 5.10488 | 0.02566 |
| gender | 0.69994 | 1 | 0.30295 | 0.58306 |
| intervention:gender | 0.10567 | 1 | 0.04574 | 0.83102 |
| Residuals | 277.24781 | 120 | NA | NA |

|  | **eta.sq** | **eta.sq.part** |
| --- | --- | --- |
| intervention | 0.04025 | 0.04080 |
| gender | 0.00239 | 0.00252 |
| intervention:gender | 0.00036 | 0.00038 |

*Moderation analyses: STAI * Nocebo effect*

|  | **Sum Sq** | **Df** | **F value** | **Pr(>F)** |
| --- | --- | --- | --- | --- |
| (Intercept) | 16.44058 | 1 | 7.70897 | 0.00617 |
| treat | 0.03902 | 1 | 0.01830 | 0.89258 |
| stai | 1.92178 | 1 | 0.90112 | 0.34395 |
| treat:stai | 0.44356 | 1 | 0.20798 | 0.64899 |
| Residuals | 332.69439 | 156 | NA | NA |

|  | **eta.sq** | **eta.sq.part** |
| --- | --- | --- |
| treat | 0.00011 | 0.00012 |
| stai | 0.00554 | 0.00574 |
| treat:stai | 0.00128 | 0.00133 |

*Moderation analyses: STAI * Intervention effect*

|  | **Sum Sq** | **Df** | **F value** | **Pr(>F)** |
| --- | --- | --- | --- | --- |
| (Intercept) | 10.85273 | 1 | 4.89887 | 0.02870 |
| intervention | 1.06105 | 1 | 0.47895 | 0.49019 |
| stai | 0.15129 | 1 | 0.06829 | 0.79427 |
| intervention:stai | 4.47274 | 1 | 2.01897 | 0.15785 |
| Residuals | 274.70393 | 124 | NA | NA |

|  | **eta.sq** | **eta.sq.part** |
| --- | --- | --- |
| intervention | 0.00358 | 0.00385 |
| stai | 0.00051 | 0.00055 |
| intervention:stai | 0.01511 | 0.01602 |

*Moderation analyses: Expectancy * Nocebo effect*

|  | **Sum Sq** | **Df** | **F value** | **Pr(>F)** |
| --- | --- | --- | --- | --- |
| (Intercept) | 31.78131 | 1 | 14.87155 | 0.00017 |
| treat | 1.82112 | 1 | 0.85216 | 0.35737 |
| expect_sideeffect | 0.99458 | 1 | 0.46540 | 0.49612 |
| treat:expect_sideeffect | 0.68426 | 1 | 0.32019 | 0.57231 |
| Residuals | 333.38048 | 156 | NA | NA |

|  | **eta.sq** | **eta.sq.part** |
| --- | --- | --- |
| treat | 0.00525 | 0.00543 |
| expect_sideeffect | 0.00287 | 0.00297 |
| treat:expect_sideeffect | 0.00197 | 0.00205 |

*Moderation analyses: Expectancy * Intervention effect*

|  | **Sum Sq** | **Df** | **F value** | **Pr(>F)** |
| --- | --- | --- | --- | --- |
| (Intercept) | 25.94094 | 1 | 11.94602 | 0.00075 |
| intervention | 23.25469 | 1 | 10.70898 | 0.00138 |
| expect_sideeffect | 3.54802 | 1 | 1.63389 | 0.20355 |
| intervention:expect_sideeffect | 9.77452 | 1 | 4.50125 | 0.03586 |
| Residuals | 269.26771 | 124 | NA | NA |

|  | **eta.sq** | **eta.sq.part** |
| --- | --- | --- |
| intervention | 0.07856 | 0.07950 |
| expect_sideeffect | 0.01199 | 0.01301 |
| intervention:expect_sideeffect | 0.03302 | 0.03503 |

*Moderation analyses: Anxiety * Nocebo effect*

|  | **Sum Sq** | **Df** | **F value** | **Pr(>F)** |
| --- | --- | --- | --- | --- |
| (Intercept) | 49.45199 | 1 | 23.16513 | 0.00000 |
| treat | 5.30536 | 1 | 2.48522 | 0.11695 |
| anxiety | 1.77175 | 1 | 0.82995 | 0.36369 |
| treat:anxiety | 0.14768 | 1 | 0.06918 | 0.79288 |
| Residuals | 333.02252 | 156 | NA | NA |

|  | **eta.sq** | **eta.sq.part** |
| --- | --- | --- |
| treat | 0.01529 | 0.01568 |
| anxiety | 0.00511 | 0.00529 |
| treat:anxiety | 0.00043 | 0.00044 |

*Moderation analyses: Anxiety * Intervention effect*

|  | **Sum Sq** | **Df** | **F value** | **Pr(>F)** |
| --- | --- | --- | --- | --- |
| (Intercept) | 50.39221 | 1 | 22.74410 | 0.00001 |
| intervention | 16.49757 | 1 | 7.44604 | 0.00728 |
| anxiety | 2.38783 | 1 | 1.07772 | 0.30123 |
| intervention:anxiety | 2.33197 | 1 | 1.05251 | 0.30693 |
| Residuals | 274.73650 | 124 | NA | NA |

|  | **eta.sq** | **eta.sq.part** |
| --- | --- | --- |
| intervention | 0.05574 | 0.05665 |
| anxiety | 0.00807 | 0.00862 |
| intervention:anxiety | 0.00788 | 0.00842 |

*Moderation analyses: Phasic EDA * Nocebo effect*

|  | **Sum Sq** | **Df** | **F value** | **Pr(>F)** |
| --- | --- | --- | --- | --- |
| (Intercept) | 63.74393 | 1 | 26.51682 | 0.00000 |
| treat | 3.41091 | 1 | 1.41890 | 0.23581 |
| phasic_mean | 0.08049 | 1 | 0.03348 | 0.85510 |
| treat:phasic_mean | 0.52625 | 1 | 0.21891 | 0.64067 |
| Residuals | 305.29600 | 127 | NA | NA |

|  | **eta.sq** | **eta.sq.part** |
| --- | --- | --- |
| treat | 0.01084 | 0.01105 |
| phasic_mean | 0.00026 | 0.00026 |
| treat:phasic_mean | 0.00167 | 0.00172 |

*Moderation analyses: Phasic EDA * Intervention effect*

|  | **Sum Sq** | **Df** | **F value** | **Pr(>F)** |
| --- | --- | --- | --- | --- |
| (Intercept) | 67.29748 | 1 | 27.44087 | 0.00000 |
| intervention | 6.65726 | 1 | 2.71453 | 0.10255 |
| phasic_mean | 0.08328 | 1 | 0.03396 | 0.85417 |
| intervention:phasic_mean | 1.60220 | 1 | 0.65330 | 0.42083 |
| Residuals | 247.69786 | 101 | NA | NA |

|  | **eta.sq** | **eta.sq.part** |
| --- | --- | --- |
| intervention | 0.02484 | 0.02617 |
| phasic_mean | 0.00031 | 0.00034 |
| intervention:phasic_mean | 0.00598 | 0.00643 |

*Moderation analyses: Tonic EDA* Nocebo effect*

|  | **Sum Sq** | **Df** | **F value** | **Pr(>F)** |
| --- | --- | --- | --- | --- |
| (Intercept) | 91.78827 | 1 | 38.23864 | 0.00000 |
| treat | 8.59044 | 1 | 3.57874 | 0.06080 |
| tonic_mean | 0.32913 | 1 | 0.13712 | 0.71178 |
| treat:tonic_mean | 0.69777 | 1 | 0.29069 | 0.59072 |
| Residuals | 304.85157 | 127 | NA | NA |

|  | **eta.sq** | **eta.sq.part** |
| --- | --- | --- |
| treat | 0.02729 | 0.02741 |
| tonic_mean | 0.00105 | 0.00108 |
| treat:tonic_mean | 0.00222 | 0.00228 |

*Moderation analyses: Tonic * Intervention effect*

|  | **Sum Sq** | **Df** | **F value** | **Pr(>F)** |
| --- | --- | --- | --- | --- |
| (Intercept) | 92.05180 | 1 | 37.46959 | 0.00000 |
| intervention | 14.34777 | 1 | 5.84024 | 0.01746 |
| tonic_mean | 0.50252 | 1 | 0.20455 | 0.65204 |
| intervention:tonic_mean | 0.41648 | 1 | 0.16953 | 0.68141 |
| Residuals | 248.12741 | 101 | NA | NA |

|  | **eta.sq** | **eta.sq.part** |
| --- | --- | --- |
| intervention | 0.05354 | 0.05466 |
| tonic_mean | 0.00188 | 0.00202 |
| intervention:tonic_mean | 0.00155 | 0.00168 |

*Moderation analyses: HRV * Nocebo effect*

|  | **Sum Sq** | **Df** | **F value** | **Pr(>F)** |
| --- | --- | --- | --- | --- |
| (Intercept) | 89.90681 | 1 | 41.98440 | 0.00000 |
| treat | 9.58692 | 1 | 4.47687 | 0.03608 |
| lf.hf_mean | 10.56606 | 1 | 4.93411 | 0.02789 |
| treat:lf.hf_mean | 2.21120 | 1 | 1.03258 | 0.31126 |
| Residuals | 308.36648 | 144 | NA | NA |

|  | **eta.sq** | **eta.sq.part** |
| --- | --- | --- |
| treat | 0.02911 | 0.03015 |
| lf.hf_mean | 0.03209 | 0.03313 |
| treat:lf.hf_mean | 0.00671 | 0.00712 |

*Moderation analyses: HRV * Intervention effect*

|  | **Sum Sq** | **Df** | **F value** | **Pr(>F)** |
| --- | --- | --- | --- | --- |
| (Intercept) | 91.05585 | 1 | 41.26568 | 0.00000 |
| intervention | 5.70098 | 1 | 2.58363 | 0.11072 |
| lf.hf_mean | 9.31627 | 1 | 4.22205 | 0.04217 |
| intervention:lf.hf_mean | 0.15499 | 1 | 0.07024 | 0.79146 |
| Residuals | 253.75618 | 115 | NA | NA |

|  | **eta.sq** | **eta.sq.part** |
| --- | --- | --- |
| intervention | 0.02029 | 0.02197 |
| lf.hf_mean | 0.03315 | 0.03541 |
| intervention:lf.hf_mean | 0.00055 | 0.00061 |

##

## **Supplementary Material 5. Cognitive performance**

## *ANOVA (Self Reported Performance on RVIP ~ Group)*

|  | **Sum Sq** | **Df** | **F value** | **Pr(>F)** |
| --- | --- | --- | --- | --- |
| (Intercept) | 231716.1987 | 1 | 526.56617 | 0.00000 |
| group | 746.8709 | 4 | 0.42431 | 0.79093 |
| Residuals | 68207.9729 | 155 | NA | NA |

|  | **eta.sq** | **eta.sq.part** |
| --- | --- | --- |
| group | 0.01083 | 0.01083 |

| **group** | **emmean** | **SE** | **df** | **lower.CL** | **upper.CL** |
| --- | --- | --- | --- | --- | --- |
| NH | 35.84 | 3.71 | 155 | 28.52 | 43.17 |
| NINSM | 36.76 | 3.65 | 155 | 29.54 | 43.97 |
| INSM | 36.75 | 3.71 | 155 | 29.42 | 44.08 |
| NISM | 41.84 | 3.77 | 155 | 34.40 | 49.28 |
| ISM | 39.12 | 3.71 | 155 | 31.80 | 46.45 |

|  | **Sum Sq** | **Df** | **F value** | **Pr(>F)** |
| --- | --- | --- | --- | --- |
| (Intercept) | 231716.19874 | 1 | 526.56617 | 0.00000 |
| treat | 196.98483 | 1 | 0.44764 | 0.50445 |
| social | 444.53419 | 1 | 1.01019 | 0.31642 |
| intervention | 59.21421 | 1 | 0.13456 | 0.71425 |
| social:intervention | 58.55667 | 1 | 0.13307 | 0.71577 |
| Residuals | 68207.97290 | 155 | NA | NA |

## *ANOVA (Estimated enhancement ~ Group)*

|  | **Sum Sq** | **Df** | **F value** | **Pr(>F)** |
| --- | --- | --- | --- | --- |
| (Intercept) | 99200.3582 | 1 | 215.25400 | 0.0000 |
| group | 702.5012 | 3 | 0.50812 | 0.6774 |
| Residuals | 57145.7176 | 124 | NA | NA |

|  | **eta.sq** | **eta.sq.part** |
| --- | --- | --- |
| group | 0.01214 | 0.01214 |

| **group** | **emmean** | **SE** | **df** | **lower.CL** | **upper.CL** |
| --- | --- | --- | --- | --- | --- |
| NINSM | 27.81818 | 3.73701 | 124 | 20.42160 | 35.21477 |
| INSM | 24.03125 | 3.79495 | 124 | 16.51998 | 31.54252 |
| NISM | 30.06452 | 3.85567 | 124 | 22.43306 | 37.69597 |
| ISM | 29.46875 | 3.79495 | 124 | 21.95748 | 36.98002 |

|  | **Sum Sq** | **Df** | **F value** | **Pr(>F)** |
| --- | --- | --- | --- | --- |
| (Intercept) | 99313.5084 | 1 | 216.92832 | 0.00000 |
| social | 472.2914 | 1 | 1.03162 | 0.31174 |
| intervention | 157.1440 | 1 | 0.34325 | 0.55902 |
| Residuals | 57227.1461 | 125 | NA | NA |

## *ANOVA (Proportion correct hits ~ group)*

|  | **Sum Sq** | **Df** | **F value** | **Pr(>F)** |
| --- | --- | --- | --- | --- |
| (Intercept) | 41.46437 | 1 | 1663.57103 | 0.00000 |
| group | 0.15271 | 4 | 1.53171 | 0.19575 |
| Residuals | 3.78859 | 152 | NA | NA |

|  | **eta.sq** | **eta.sq.part** |
| --- | --- | --- |
| group | 0.03875 | 0.03875 |

| **group** | **emmean** | **SE** | **df** | **lower.CL** | **upper.CL** |
| --- | --- | --- | --- | --- | --- |
| NH | 0.57018 | 0.02882 | 152 | 0.51323 | 0.62712 |
| NINSM | 0.50159 | 0.02748 | 152 | 0.44730 | 0.55589 |
| INSM | 0.50329 | 0.02791 | 152 | 0.44815 | 0.55843 |
| NISM | 0.52105 | 0.02882 | 152 | 0.46410 | 0.57800 |
| ISM | 0.47533 | 0.02791 | 152 | 0.42019 | 0.53047 |

|  | **Sum Sq** | **Df** | **F value** | **Pr(>F)** |
| --- | --- | --- | --- | --- |
| (Intercept) | 41.46437 | 1 | 1663.57103 | 0.00000 |
| treat | 0.11840 | 1 | 4.75046 | 0.03083 |
| social | 0.00057 | 1 | 0.02300 | 0.87967 |
| intervention | 0.01537 | 1 | 0.61660 | 0.43353 |
| social:intervention | 0.01783 | 1 | 0.71518 | 0.39906 |
| Residuals | 3.78859 | 152 | NA | NA |

|  | **eta.sq** | **eta.sq.part** |
| --- | --- | --- |
| treat | 0.03004 | 0.03031 |
| social | 0.00015 | 0.00015 |
| intervention | 0.00390 | 0.00404 |
| social:intervention | 0.00452 | 0.00468 |

## **Supplementary Material 6. Physiological Measures**

*Materials and measures*

*Equivital Sensor Belt and Module*. Participant Heart Rate (HR) was measured using an Equivital Sensor Module fitted onto an Equivital Sensor Belt. HR was measured continuously for 15 minutes after administration of the treatment (or lack thereof). HR Variability (HRV) is measure of the parasympathetic and sympathetic branches that modulate cardiac activity ^[1]^. Spectral analysis was used to investigate the High Frequency/Low Frequency (HF/LF) ratio using the pyHRV package ^[2]^.

*Electrodermal activity (EDA).* EDA was recorded as a measure of autonomic arousal in response to the treatment and social communication manipulations. This was achieved by using a PowerLab DAQ and Galvanic Skin Response amplifier (ADInstruments) with two electrodes placed on the middle and index fingers of participants’ right hand. EDA was recorded over the same time period as HR. EDA can be decomposed into tonic activity (slower general changes in sympathetic arousal) and phasic activity (reactive fast changes), which each reflect important components of physiological arousal ^[3]^. A convex optimization approach to electrodermal activity was applied to extract the two components ^[4]^, after which mean Skin Conductance Level ($\mu$SCL) was calculated as a measure of the tonic activity and the Skin Conductance Response (SCR) as a measure of the phasic activity.

*Results*

*HRV*. Due to technical issues with the Equivital Sensor Module, complete heart rate data was available for 148 participants ($N_{Missing}$=12). There was no significant relationship between missing data and participant group, $\chi_{4}^{2}$=0.48, *p*=.98, Cramer’s V=.05. Three-way mixed 2(Time: first five minutes vs second five minutes, where five minutes is the time point side effect modelling occurred in groups that received modelling) x 2(Induction Method) x 2(Intervention) + 1(Natural History) ANOVA was conducted to explore differences in HRV. There was no significant effect of Time, Induction Method, Intervention, nor interactions, all *p*>.060.

*EDA*. Due to technical issues, complete EDA recordings for 131 participants were available ($N_{Missing}$=29). There was no significant relationship between missing data and participant group, $\chi_{4}^{2}$=4.75, *p*=.31, Cramer’s V=.17. Three-way mixed 2(Time) x 2(Induction Method) x 2(Intervention) + 1(Natural History) ANOVA was conducted to explore differences in tonic and phasic activity, separately. Holding the other factors constant, there was a significant interaction between treatment and time on Tonic activity, such that there was a larger increase in tonic activity in treatment groups than the Natural History group. With respect to phasic activity, there was a main effect of time such that the second time period (i.e., after social modelling in the social modelling groups) had higher phasic activity than the first. There was a main effect of the intervention, such that those who received the intervention had reduced phasic activity than those who did not. No other effects reached significance, all *p*>.066.

*EDA ANOVA Contrasts*

|  | Tonic (μSCL) | | | Phasic (SCR) | | |
| --- | --- | --- | --- | --- | --- | --- |
|  | *t*(133) | *p* | ${\eta_{p}}^{2}$ | *t*(133) | *p* | ${\eta_{p}}^{2}$ |
| Time | 0.835 | 0.405 | 0.005 | 2.016 | 0.046 | 0.030 |
| Treatment | 0.109 | 0.914 | 0.000 | 0.440 | 0.660 | 0.001 |
| Intervention | 1.422 | 0.157 | 0.015 | 2.213 | 0.029 | 0.036 |
| Induction Method | -1.625 | 0.107 | 0.019 | 0.525 | 0.600 | 0.002 |
| Treatment*Time | -1.668 | 0.098 | 0.020 | -0.942 | 0.348 | 0.007 |
| Intervention*Time | -2.029 | 0.044 | 0.030 | -0.355 | 0.723 | 0.001 |
| Induction Method*Time | 1.300 | 0.196 | 0.013 | 0.470 | 0.639 | 0.002 |
| Intervention*  Induction Method* Time | 0.859 | 0.392 | 0.006 | 1.855 | 0.066 | 0.025 |

### *Group Means (Tonic EDA)*

| **group** | **name** | **emmean** | **SE** | **df** | **lower.CL** | **upper.CL** |
| --- | --- | --- | --- | --- | --- | --- |
| nh | tonic_BW | 0.157 | 1.433 | 133 | -2.677 | 2.991 |
| ninsm | tonic_BW | 5.054 | 1.489 | 133 | 2.108 | 7.999 |
| nism | tonic_BW | 3.670 | 1.383 | 133 | 0.935 | 6.405 |
| insm | tonic_BW | 0.820 | 1.383 | 133 | -1.914 | 3.555 |
| ism | tonic_BW | 3.222 | 1.407 | 133 | 0.439 | 6.005 |
| nh | tonic_AW | 0.634 | 2.033 | 133 | -3.386 | 4.655 |
| ninsm | tonic_AW | 5.685 | 2.112 | 133 | 1.506 | 9.863 |
| nism | tonic_AW | 5.195 | 1.961 | 133 | 1.315 | 9.074 |
| insm | tonic_AW | -0.358 | 1.961 | 133 | -4.237 | 3.522 |
| ism | tonic_AW | 2.872 | 1.996 | 133 | -1.076 | 6.820 |

### *Group Means Phasic EDA*

| **group** | **name** | **emmean** | **SE** | **df** | **lower.CL** | **upper.CL** |
| --- | --- | --- | --- | --- | --- | --- |
| nh | phasic_BW | 0.622 | 0.177 | 133 | 0.272 | 0.971 |
| ninsm | phasic_BW | 0.865 | 0.184 | 133 | 0.502 | 1.228 |
| nism | phasic_BW | 0.709 | 0.171 | 133 | 0.372 | 1.047 |
| insm | phasic_BW | 0.432 | 0.171 | 133 | 0.095 | 0.769 |
| ism | phasic_BW | 0.606 | 0.174 | 133 | 0.262 | 0.949 |
| nh | phasic_AW | 0.966 | 0.236 | 133 | 0.499 | 1.433 |
| ninsm | phasic_AW | 0.936 | 0.245 | 133 | 0.451 | 1.421 |
| nism | phasic_AW | 0.955 | 0.228 | 133 | 0.505 | 1.406 |
| insm | phasic_AW | 0.698 | 0.228 | 133 | 0.247 | 1.148 |
| ism | phasic_AW | 1.103 | 0.232 | 133 | 0.645 | 1.562 |

**References**

1. Daniali H, Flaten MA. Placebo Analgesia, Nocebo Hyperalgesia, and the Cardiovascular System: A Qualitative Systematic Review. *Front Physiol*. 2020;11:549807. doi:10.3389/fphys.2020.549807

2. Gomes P, Margaritoff P, Silva H. pyHRV: Development and evaluation of an open-source python toolbox for heart rate variability (HRV). 2019:822-828.

3. Braithwaite JJ, Watson DG, Jones R, Rowe M. A guide for analysing electrodermal activity (EDA) & skin conductance responses (SCRs) for psychological experiments. *Psychophysiology*. 2013;49(1):1017-1034.

4. Greco A, Valenza G, Lanata A, Scilingo EP, Citi L. cvxEDA: A Convex Optimization Approach to Electrodermal Activity Processing. *IEEE Trans Biomed Eng*. Apr 2016;63(4):797-804. doi:10.1109/tbme.2015.2474131
